# Lower respiratory tract microbiome composition and community interactions in smokers

**DOI:** 10.1099/acmi.0.000497.v3

**Published:** 2023-03-21

**Authors:** Michael Campos, Trevor Cickovski, Mitch Fernandez, Melita Jaric, Adam Wanner, Gregory Holt, Elio Donna, Eliana Mendes, Eugenia Silva-Herzog, Lisa Schneper, Jonathan Segal, David Moraga Amador, Juan Daniel Riveros, Vanessa Aguiar-Pulido, Santanu Banerjee, Matthias Salathe, Kalai Mathee, Giri Narasimhan

**Affiliations:** ^1^​ Division of Pulmonary, Allergy, Critical Care and Sleep Medicine, Miller School of Medicine, University of Miami, Miami, FL, USA; ^2^​ Bioinformatics Research Group (BioRG), School of Computing and Information Sciences, Florida International University, Miami, FL, USA; ^3^​ Department of Molecular Microbiology and Infectious Diseases, Department Human and Molecular Genetics, Herbert Wertheim College of Medicine, Florida International University, Miami, FL, USA; ^4^​ Interdisciplinary Center for Biotechnology Research, University of Florida, Gainesville, FL, USA; ^5^​ Department of Surgery, Miller School of Medicine, University of Miami, Miami, FL, USA; ^6^​ Department of Internal Medicine, University of Kansas Medical Center, Kansas City, KS 66160, USA; ^7^​ Florida International University, Biomolecular Sciences Institute, Miami, FL, USA

**Keywords:** co-occurrence networks, clustering, lower respiratory tract (LRT), microbiome, smoking

## Abstract

The lung microbiome impacts on lung function, making any smoking-induced changes in the lung microbiome potentially significant. The complex co-occurrence and co-avoidance patterns between the bacterial taxa in the lower respiratory tract (LRT) microbiome were explored for a cohort of active (AS), former (FS) and never (NS) smokers. Bronchoalveolar lavages (BALs) were collected from 55 volunteer subjects (9 NS, 24 FS and 22 AS). The LRT microbiome composition was assessed using 16S rRNA amplicon sequencing. Identification of differentially abundant taxa and co-occurrence patterns, discriminant analysis and biomarker inferences were performed. The data show that smoking results in a loss in the diversity of the LRT microbiome, change in the co-occurrence patterns and a weakening of the tight community structure present in healthy microbiomes. The increased abundance of the genus *Ralstonia* in the lung microbiomes of both former and active smokers is significant. Partial least square discriminant and DESeq2 analyses suggested a compositional difference between the cohorts in the LRT microbiome. The groups were sufficiently distinct from each other to suggest that cessation of smoking may not be sufficient for the lung microbiota to return to a similar composition to that of NS. The linear discriminant analysis effect size (LEfSe) analyses identified several bacterial taxa as potential biomarkers of smoking status. Network-based clustering analysis highlighted different co-occurring and co-avoiding microbial taxa in the three groups. The analysis found a cluster of bacterial taxa that co-occur in smokers and non-smokers alike. The clusters exhibited tighter and more significant associations in NS compared to FS and AS. Higher degree of rivalry between clusters was observed in the AS. The groups were sufficiently distinct from each other to suggest that cessation of smoking may not be sufficient for the lung microbiota to return to a similar composition to that of NS.

## Data Summary

Raw sequence files and abundance files (with metadata information on sample labels) for all samples used in this study can be downloaded currently from the BioRG website (http://biorg.cs.fiu.edu/Smoking/). We have deposited our data into NCBI (Accession Number: PRJNA880638). Our entire downstream analysis is available open-source as a PluMA pipeline (http://biorg.cs.fiu.edu/pluma/pipelines.html).

## Introduction

Smoking, a known contributor to the development of human disease, can affect the resident microbial communities in different body niches [1]. Several mechanisms have been proposed, including smoking-related immunosuppression [2], enhancement of biofilm formation for certain species [3, 4] and species selection by the effect of local oxygen tension [1]. Further, the oral cavities and upper airways have direct contact with smoking chemicals, heat and the microbes contained in cigarettes, all of which can play a role in altering the microbiome composition [1].

Therefore, it is not surprising that the microbial profiles of the oral and nasopharyngeal regions showed marked differences between active (AS), former (FS) and never smokers (NS) in previous studies [5]. It has been postulated that the smoking-related dysbiosis in the oral microbiome may account for the increased prevalence of respiratory tract complications seen in smokers [6]. Significant differences in the composition of the subgingival microbiome in periodontitis have been described between smokers and nonsmokers [7]. The salivary microbial signature at the genera level is in fact so well differentiated that biomarkers discovered using linear discriminant analysis effect size (LEfSe) can be used to classify smokers and nonsmokers [8]. These differences may change with smoking cessation, as the significant differences in weighted UniFrac, unweighted UniFrac and Bray–Curtis distances observed in the overall tongue microbiome compositions between current and never smokers are not seen between former and never smokers [9]. The effects of smoking on the lower respiratory tract (LRT) microbiome are less clear.

It is now well established that the LRT is not sterile and harbours distinct microbiota from the upper respiratory tract [10–12]. A significant portion of the healthy lung microbiome is similar to that of the oral and nasopharyngeal cavities, most likely by microaspiration [13, 14]. Accordingly, studies using bronchoalveolar lavage (BAL) sampling have shown that the LRT microbial communities in healthy individuals are dominated by bacteria from the phyla Firmicutes, Proteobacteria, Actinobacteria and Bacteroidetes [15]. Unique LRT-specific organisms occur at very low levels but are variable between individuals and may change regionally depending on the lung microarchitecture [13, 16, 17]. Studies on the LRT bacterial composition in cases of smoking-associated diseases such as chronic obstructive pulmonary disease (COPD) [16, 18–20], pulmonary fibrosis [21] and lung cancer [22, 23] have shown distinct differences compared with non-diseased subjects. It is therefore possible that microbial dysbiosis affects the pathogenesis of the disease [24, 25], which highlights that a more in-depth understanding of the effects of smoking on the resident microbial communities of the lung is needed.

The influence of smoking on the LRT microbial composition was studied by Morris *et al.* [25]. Using BAL samples from 45 nonsmokers and 19 current smokers, they described significant differences in the oral microbial communities of nonsmokers compared with smokers, but their analysis did not show significant differences in the lung microbiomes of the two cohorts [25]. The investigation by Morris *et al.* was confined to surveying the differential abundances of various bacterial taxa. Further, their study did not include any former smokers. In this study, we sought to compare the LRT microbiome profiles of AS, FS and NS using standard ecological parameters and analytical tools to better characterize the bacterial communities of the lung. By assuming that frequently interacting bacterial taxa must co-occur, we used co-occurrence analysis to obtain a glimpse into the possible ‘social network’ [26] among the bacterial taxa in the lung microbiome.

## Methods

### Participant selection

The study presented here is a prospective single-centre cohort study approved by the University of Miami’s Institutional Review Board. Volunteer subjects had to be >40 years old, either nonsmokers or smokers of at least 10 pack-years in their lifetime. Former smokers had to be abstinent of tobacco use for at least 12 months, whereas active smokers had to have smoked at least one cigarette within 3 days of enrolment. Participation exclusion criteria included pulmonary malignancies or prior thoracic surgery, use of antibiotics, systemic steroids or immunosuppressant agents within 12 weeks, chronic use of inhaled medications for any lung condition, or presence of acute upper respiratory infection within the previous 4 weeks. In short, participation exclusion criteria included the diagnosis of chronic lung disease with the exception of COPD (including chronic bronchitis). Note that the definitions of COPD and chronic bronchitis used for clinical purposes are arbitrary and therefore we did not exclude subjects with these clinical diagnoses because the intention of the study was to assess the interaction of smoking and the microbiome. In addition, we made sure that subjects were in a good clinical condition to undergo a bronchoscopy as per the physician investigators.

All subjects underwent complete pulmonary function testing and a detailed clinical and demographic questionnaire. The sampling procedure was standardized for all subjects, using a flexible bronchoscope (Olympus, PA, USA) via the nasal passage under moderate sedation in accordance with standard clinical practice. After the anaesthetization of vocal cords and trachea with 1 % lidocaine, the bronchoscope was directly inserted into the right middle lobe bronchus and wedged. Bronchoalveolar lavage fluid (BAL) was collected by instilling three 20 ml aliquots of normal saline, immediately aspirated using a collector trap inserted on the suction port of the bronchoscope. The BAL was then centrifuged, and the supernatant stored in 1 ml aliquots at −80 °C until analysis.

### DNA purification

Total DNA was extracted from the BAL samples using a FastDNA SPIN kit for soil (MP Biomedicals, Solon, OH, USA) with a slightly modified protocol. Briefly, the BAL samples were thawed, added to Lysing Matrix E tubes, and homogenized in the FastPrep (MP Biomedicals, Solon, OH, USA) instrument. After centrifugation and supernatant separation, the protein was precipitated out using protein precipitation solution (PPS). To isolate the metagenomic DNA, the Binding Matrix solution was added to the supernatant. The mixture was then added to a SPIN Filter and Catch Tube apparatus and centrifuged. The contents of the catch tube were discarded. The column was washed with salt/ethanol wash solution (SEWS-M), centrifuged and air-dried for 5 min. The metagenomic DNA was eluted using 100 µl of DNase/pyrogen-free water (DES). To increase DNA yield, the tube was incubated for 5 min at 55 ˚C in a heat block before centrifugation. The DNA was quantified via UV spectrophotometry on the Synergy HT Multi-Detection Microplate Reader (BioTek Corporation, Winooski, VT, USA).

### PCR amplification of regions of the 16S rRNA gene

A single PCR amplification was performed to amplify the V6–V8 region of the 16S rRNA gene from BAL sample metagenomic DNA extracts. The choice of primers and the region to be amplified were optimized previously [27]. The degenerate primer pairs that were utilized in the amplification were MJ_2_68_F (5′- TGCATGGWWGTCGTCAGC-3′), MJ_2_68_R (5′-TGTGTACAAGWCCCGWGAACG-3′). Final concentrations and volumes of the reaction mixture were 2 µl of 10× PCR buffer (15 mM MgCl_2_) (Qiagen, Venlo, Netherlands), 0.4 µl of 10 µM dNTPs, 0.4 µl of HPLC-purified forward and reverse primers (10 µM), 0.06 µl HotStarTaq *Plus* (Qiagen), 1 µl of 10 ng µl^−1^ DNA template and 16 µl of PCR-grade water.

All amplifications were performed on either a PTC-200 Peltier Thermal Cycler (MJ Research, Waltham, MA, USA) or a Touchgene Gradient PCR Thermal Cycler (Techne, Burlington, NJ, USA). The amplification parameters used were as follows: 95 °C for 5 min initial denaturation and HotStarTaq *Plus* activation, followed by 35 cycles of denaturation at 95 °C for 20 s, primer annealing at 50 °C for 1 min and an extension at 72 °C for 1 min, followed by a final elongation step at 72 °C for 10 min. Total DNA from *Pseudomonas aeruginosa* PAO1 was used as a positive control. PCR reactions without the template served as a negative control. The PCR amplifications were run either in duplicate or triplicate, depending on the DNA yield.

### Gel electrophoresis and DNA purification

Gel electrophoresis was performed using a 2 % (weight/volume) 0.5× Tris–Borate–EDTA (TBE) agarose gel (MG Scientific JT Baker Agarose, Pleasant Prairie, WI, USA). A 100 bp DNA ladder was co-loaded as a size reference. Gels were stained with Invitrogen SYBR Safe DNA Gel Stain (Life Technologies, Grand Island, NY, USA) and viewed under UV trans-illumination on a KODAK Gel Logic 2200 Imaging System (Carestream Health, Rochester, NY, USA). The appropriate bands were excised and purified using the Wizard SV Gel and PCR Clean-Up System (Promega, Madison, WI, USA) and the provided protocol. In brief, the gel slices were melted and dissolved in Membrane Binding Solution (Promega, Madison, WI) and then added to an SV Minicolumn assembly and centrifuged. The bound DNA was washed several times using Membrane Wash Solution and then eluted using 50 µl of nuclease-free water.

### 454 sequencing

#### Library construction

The resulting amplicons were treated as shotgun fragments for library construction (ICBR Core Facilities, University of Florida, Gainesville, FL, USA). The protocol used was a slight modification of the method described in the user’s manual provided with the Rapid Library Construction kit (Roche Applied Science, Penzberg, Germany; Rapid Library Preparation Manual, GS FLX Titanium Series, October 2009, revised January 2010). The final library was quantitated in the QUBIT fluorometer (Invitrogen/Thermo Fisher, Waltham, MA, USA) and the BioAnalyzer (Agilent, Santa Clara, CA, USA). The conditions and procedures for emulsion PCR and bead enrichment are as described in the Roche protocols. A total of 340 000 enriched sequencing beads were loaded on a 1/8 region of a picotitre plate for sequencing on the Roche 454 GS FLX instrument.

#### Image processing

Raw image signals were processed offline using the GS Run Browser application that resides within the computer connected to the instrument (software version 2.0). Amplicon libraries were prepared using the ‘standard’ signal-processing pipeline. The process includes a series of normalization, correction and quality-filtering algorithms, and the application threads the remaining (high-quality) signals into ‘flowgrams’ for each well (reads). Base calls with associated quality scores for the individual reads were produced and output as standard flow gram format files. A library sample that was successfully sequenced on a 1/8 picotitre plate region (Titanium Chemistry) typically yielded 70 000–100 000 reads, 300–400 bp long, and an average quality score of ≥30.

### Operational taxonomic units (OTUs)

A basic workflow was modelled on Costello stool analysis [28]. An oligos file was written manually for each sequencing pool to group reads by subject. Pools contained a maximum of 12 samples with each group (AS, FS and NS) randomly distributed among the pools, and each sample with a unique barcode. One base error was allowed per barcode. Primers were not removed from sequences. Cutoffs were set at 25 for the quality score and 50 bp for minimum length. Sequences were aligned using the Silva Bacteria Alignment Database as a reference [29] and the Needleman–Wunsch alignment algorithm with a k-mer size of 8 [30]. The reverse complement was used for the alignment if 50 % or more of the reads were removed during the original alignment, indicating that the reads may have been flipped. The chimaeras were removed using UCHIME [31]. The classification was performed with a k-mer size of 7 and a confidence cutoff of 60. Reads were clustered into OTUs based on sequence similarity (distance <0.03%) and classified against the silva database (v. 132) [29]. Each read for each subject was mapped to an OTU. Abundance for each OTU for each subject was measured as the number of reads mapped to that particular OTU.

#### Decontamination

Reads below 99 % confidence at the kingdom level (bacteria or archaea) were considered to be contaminated and removed, as were all eukaryotes, mitochondria, archaea and chloroplasts. An early analysis of read counts indicated contamination by members of the genus *Halomonas *. This was repeatedly confirmed by PCR performed in saline washes of the sterilized bronchoscopes (data not shown). Reads mapped to the *Halomonas* genome were manually removed from further analysis.

#### Normalization

The raw read counts were normalized by scaling them to the range 0 through 1, which was achieved by dividing each entry by the sum of all the entries for that subject. For each group, OTUs with non-zero normalized counts in less than 50 % of the members were eliminated. This means that the set of OTUs retained could be different for each group. As this would create bias in comparative and biomarker analyses (LEfSe, DESeq2, PLSDA), we kept OTUs with non-zero normalized counts in more than 50 % of one of the groups in all groups for those analyses, creating a uniform OTU set.

### Bioinformatic analyses

#### Compositional analyses

Alpha diversity was measured using Chao richness [32] and inverse Simpson diversity [33] indices. The DESeq2 [34] algorithm was used to find distinguishing taxa (based on abundance) for each group.

#### Biomarker analyses

The linear discriminant analysis effect size or LEfSe analysis [35] was used for the identification of potential biomarkers (*P*<0.05, LDA effect size >1) to produce a set of taxa with relative abundances most significantly different between each group and the other two. This analysis determines the organisms and clades that best differentiate the groups under study (AS, FS, NS) by combining effect relevance with biological consistency. By providing an effect size, this method can also rank the significance of the biomarkers (i.e. taxa). Finally, the visualizations provided by LEfSe places these biomarkers on taxonomic trees, allowing for improved biological interpretations. LEfSe can be especially useful for discovering differences in taxa with relatively low relative abundance, and that is not necessarily apparent in the compositional bar graph.

#### Clustering analyses

Principal coordinate analysis (PCoA) [36] was used to analyse overall data clustering, with unweighted Unifrac as a measure of beta diversity within and between cohorts. Three-dimensional visualizations of the results were produced using Emperor (0.9) and ggplot2 (3.3.3). Partial least squares differential analysis (PLS-DA [37]) was used to determine the degree of separation between the three cohorts of samples. Markov clustering (MCL [38]) was used to cluster nodes in the CoN, and these same clusters were used in the heatmaps to illustrate tightness.

#### Network analyses

Proclivities of OTUs to co-occur in each of the subject groups were investigated using pairwise correlations computed using SparCC (*P*<0.05) [39]. SparCC iteratively estimates correlation using log ratio transformations of relative abundances, and operates under the assumption that microbial data are sparse. Network diagrams were constructed from pairwise correlation matrices using Cytoscape [40] to better visualize the complex interactions between OTUs in each of the subject groups. Node sizes were set to reflect the relative abundance of each OTU. For each group, the correlation coefficients were calculated for each pair of OTUs, and the thickness of the edge was set to reflect the magnitude of the correlation value. The resulting matrix was then represented by a network that integrated many vital pieces of information in a single plot to help visualize relationships between OTUs present in each specific group using Cytoscape [40]*,* and were visualized using the Fruchterman–Reingold algorithm [41].

## Results

### Cohort

The basic demographic profiles of the 55 subjects enrolled are shown in Table 1. All groups had similar distributions for age, gender and lung function parameters. All 46 smokers had the same smoking exposure in pack-years, with the FS quitting an average of 10.2±9.7 years before participation (range 1.5 to 34 years). As expected, the smoker populations, FS and AS, have reduced forced vital capacity (FVC), forced expiratory volume at second 1 (FEV_1_) and diffusing capacity for carbon monoxide (D_L_CO), but the differences were not significant by analysis of variance (ANOVA) (data not shown). The 24 FS and the 22 AS differed significantly (χ^2^ test, *P*<0.05) only in a higher prevalence of clinical chronic bronchitis in the AS group. Where appropriate, values are provided as an average value ±standard deviation along with the median value within square brackets.

**Table 1.** Clinical and demographic characteristics of the study cohort. All results are shown as mean±sd [median value] or *n* (%)

	Total/average	Never smokers	Smokers by smoking status	
			Former	Active
**No. of subjects**	55	9	24	22
**Age**	54.4±10.4	56.0±13.5	55.4±10.3	52.7±9.5
**Male (%)**	33 (60.0)	5 (55.5)	13 (54.1)	15 (68.1)
**Pack-years**	38.9±29.4	0	38.5±34.7	39.3±23.0
**FVC (L)**	3.6±0.8	3.8±0.7 [3.7]	3.6±0.9 [3.6]	3.7±0.8 [3.4]
**FVC (%)**	92.4±17.0	96.8±10.9 [94.0]	91.8±20.4 [89.0]	91.2±15.3 [87.0]
**FEV1 (L)**	2.7±0.8	3.1±0.5 [2.7]	2.6±0.8 [2.5]	2.7±0.8 [2.6]
**FEV1 (%)**	87.8±23.2	100.4±15.5 [98.0]	85.0±25.1 [83.5]	85.8±22.8 [84.0]
**FEV1/FVC**	74.4±11.9	81.7±6.7 [84.8]	72.8±12.3 [76.0]	73.1±12.4 [78.5]
**DLCO (%)**	90.5±22.3	100.6±20.0 [97.0]	94.1±25.6 [93.0]	82.4±16.8 [79.0]
**Chronic bronchitis (%)**	14 (25.4)	0	3 (12.5)	11 (50)*

*P<0.05 compared with the former smoker group.

DLCO, diffusing capacity for carbon monoxide; FEV, forced expiratory volume; FVC, forced vital capacity.

### Microbial abundance and smoking status

On average, more than 3600 reads with an average length of 479 nucleotides were recorded in each subject’s BAL, which allowed the characterization of approximately 400 OTUs per subject. Table 2 shows the statistics for each group, including the number of OTUs recovered, unclassifiable reads (k-mer size=7, confidence cutoff=60, cluster sequence similarity distance cutoff=0.03 %), and richness and diversity indices.

**Table 2.** Sequencing outputs, richness and diversity indices

	ALL	Never smokers	Former smokers	Active smokers
Total reads	3648.6±3009.8	2551.6±2048.6	3628.6±3105.5	4119.2±3222.7
OTUs recovered	29.3±2.2	54.8±7.9	23.3±1.8	25.2±1.4
Reads unclassified at genus level	1051.3±125.6	1489.0±328.0	1250.2±207.6	636.4±132.3
Richness (Chao)	36.2±3.0	71.9±11.2	26.2±1.8	32.3±1.8
Diversity (inverse Simpson index)	4.4±0.4	7.6±1.5	3.4±0.4	4.3±0.6

**Fig. 1.**
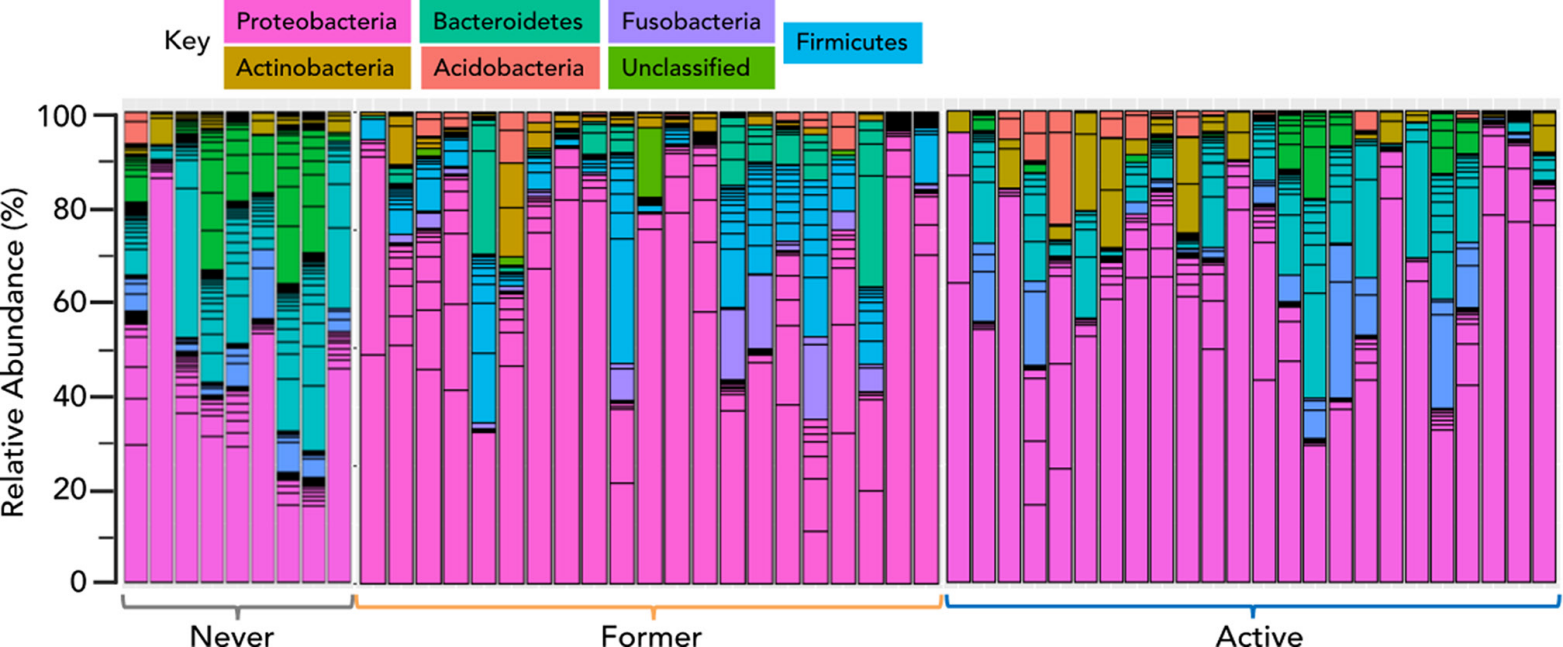
Relative abundance at the level of phyla. The relative abundance was plotted for never smokers, former smokers and active smokers at the phylum level. The columns correspond to the subjects studied.

The LRT compositional profile in all three study groups, namely NS, AS and FS, is represented in Fig. 1. The NS profile is fairly balanced between the dominant phyla Proteobacteria (39 %), Firmicutes (31 %) and Bacteroides (21 %), followed by Fusobacteria and Actinobacteria with relative abundance in the single digits (7 and 2 %, respectively). The FS group showed a marked increase in Proteobacteria (61 %) at the expense of Firmicutes (19 %) and Bacteroides (dropping all the way to 4 %, even lower than Fusobacteria). This trend is also true in the active smokers, with Proteobacteria composing an overwhelming 75 % and Firmicutes dropping to 11 %.

**Fig. 2.**
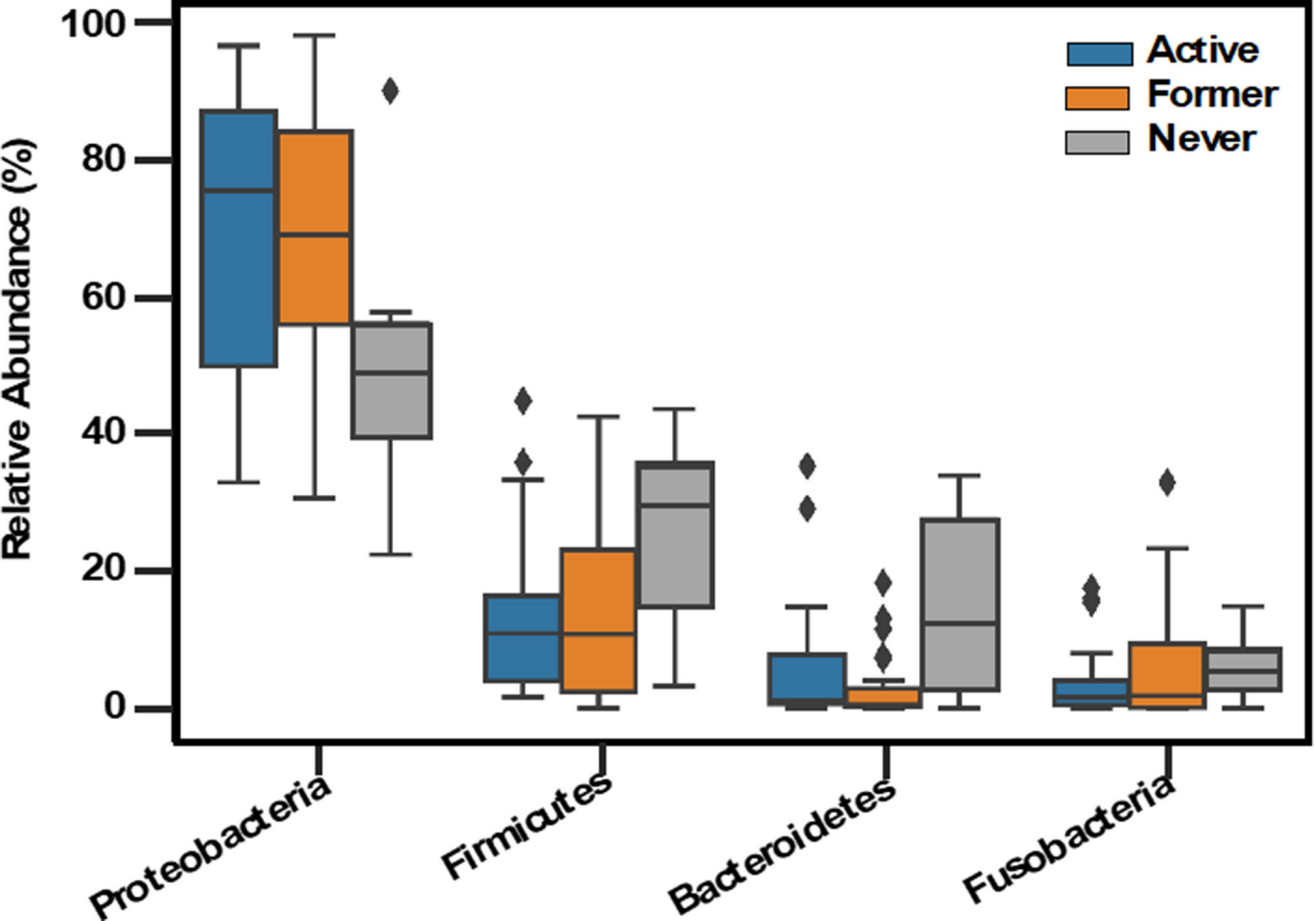
Comparisons of relative abundances of dominant phyla. Box-and-whisker plot of the relative abundances of the four most dominant (based on mean relative abundance) phyla in active, former and never smoker lung microbiomes.

Fig. 2 shows box-and-whisker plots summarizing the relative abundance of the four most abundant phyla in all the samples (note that outliers are included).

**Fig. 3.**
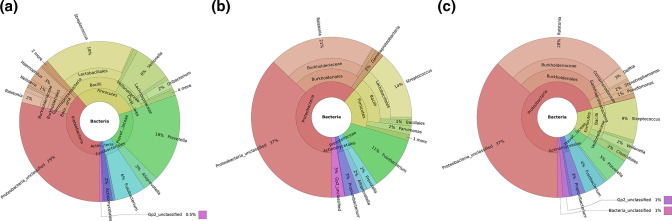
Krona plots showing the taxonomic organization of the abundance data. Significant abundance data and distribution displayed using Krona in never smokers (**a**), former smokers (**b**) and active smokers (**c**). Taxonomy nodes are shown as nested sectors arranged from the top level of the hierarchy at the centre and progressing outward.

The distribution of recovered OTUs for each subject group is visualized in Fig. 3. These Krona images allow intuitive exploration of relative abundances within the complex hierarchies of metagenomic classifications [42]. The genus-level analyses (Fig. 3) show that most of the increase in Proteobacteria in FS and AS compared to its abundance in NS was contributed by the genus *Ralstonia *, which increased from 2 % (NS) to 21 % in FS and 28 % in AS. From the phylum Firmicutes, the genus *Streptococcus* (reduced from 18 % in NS to 14 and 9 % in FS and AS, respectively) and the genus *Veillonella* (reduced from 8 % in NS to 1 and 2 % in FS and AS, respectively), in addition to the genus *Prevotella* from the phylum Bacteroidetes (15 % in NS to 2 and 5 % in FS and AS, respectively), were the taxa with the highest reductions in relative abundance. Each of these are known members of healthy lung microbiota [11]. The genus *Propionibacterium,* from the phylum Actinobacteria, showed a small increase from 0.8 % in NS to 3 % in FS and AS, respectively.

Using DESeq2 [34] we conducted a pairwise comparison of individual OTUs to determine OTUs found to be significantly more abundant in each subject group over one or both other groups (*P*<0.05), summarized in Table 3. *P*-values were corrected for multiple comparisons using the Benjamini–Hochberg method [43]. Each column is labelled by a cohort (NS, FS, AS), and contains a list of the taxa that are differentially abundant in that cohort. Interquartile range calculations are performed for each sample based on taxa relative abundance, and taxa are subsequently ordered by their median quartile over all samples in the corresponding cohort. *Ralstonia *.(01–03) were upper-quartile taxa in the AS and FS profiles that were differentially abundant when compared to NS in both profiles, offering further support for the observed behaviour in the Krona plots, and also that dominant Proteobacteria was a distinguishing characteristic of AS and FS compared to NS. A subsequent run of DESeq2 at the phylum level did turn up Proteobacteria as distinguishing AS and NS (but interestingly, not FS and NS), along with Acidobacteria. Additionally, two other top-quartile Proteobacteria (*Burkholderia.*01–02) also distinguished AS from NS. When viewing the NS profile, far more upper-quartile taxa were found as distinguishing from AS [including a *Streptococcus *, *Fusobacterium *, *Veillonella* (distinguished from both other groups) and *Haemophilus *], while the lower-quartile taxa tended to distinguish from FS. The three top-quartile NS taxa: *Streptococcus.*03, *Fusobacterium.*02 and *Veillonella.*03 were also discovered by ANCOM with bias correction (ANCOM-BC [44]) as distinguishing for NS (it did not uncover any as distinguishing for FS or AS). The NS profile had a total of eight taxa that have previously been reported as members of healthy oral [45] and lung [11] microbiomes. The FS profile had two of these taxa, and both distinguished from AS. AS had one distinguishing from FS.

**Table 3.** Genera that were found to be significantly more abundant in each of the study groups using DESeq2. For each column, taxa differentially abundant with respect to one other group have the second group provided in parentheses (taxa differentially abundant with respect to both the remaining groups do not have any cohorts mentioned in parentheses and are marked in bold. Highlighted taxa distinguish two different groups from the same third group

Quartile	Never smokers (NS)	Former smokers (FS)	Active smokers (AS)
**1**	* Streptococcus *.03*† (AS)	*Ralstonia.*01 (NS)	*Ralstonia.*01 (NS)
	*Fusobacterium.*02* (AS)	*Ralstonia.*02 (NS)	*Ralstonia.*02 (NS)
	*Veillonella.*03*^+^	* Ralstonia* 03 (NS)	*Pelomonas.*01 (NS)
	*Haemophilus.*03* (AS)		*Prevotella.*06*† (FS)
			*Burkholderia.*01 (NS)
			*Burkholderia.*02 (NS)
**2**	*Prevotella.*18*† (FS)		*Stomatobaculum.*01 (FS)
	*Prevotella.*06* ^†^ (FS)		*Ralstonia.*03 (NS)
**3**	*Prevotella.*03*†	*Neisseria.*01* (AS)	***Delftia.*01**
	*Leptotrichia.*01* (FS)		*Strenotrophomonas.*01 (NS)
	*Methylobacterium.*01 (AS)		
**4**	*Lautropia.*01 (FS)	*Fusobacterium.*02* (AS)	

*Microbes with species and strains associated with normal oral flora [45].

†Microbes with species and strains associated with normal lung flora [11].

AS, active smoker; blue, FS/NS vs AS; FS, former smoker; NS, never smoker; Pink, AS/FS vs NS; yellow, AS/NS vs FS.

**Fig. 4.**
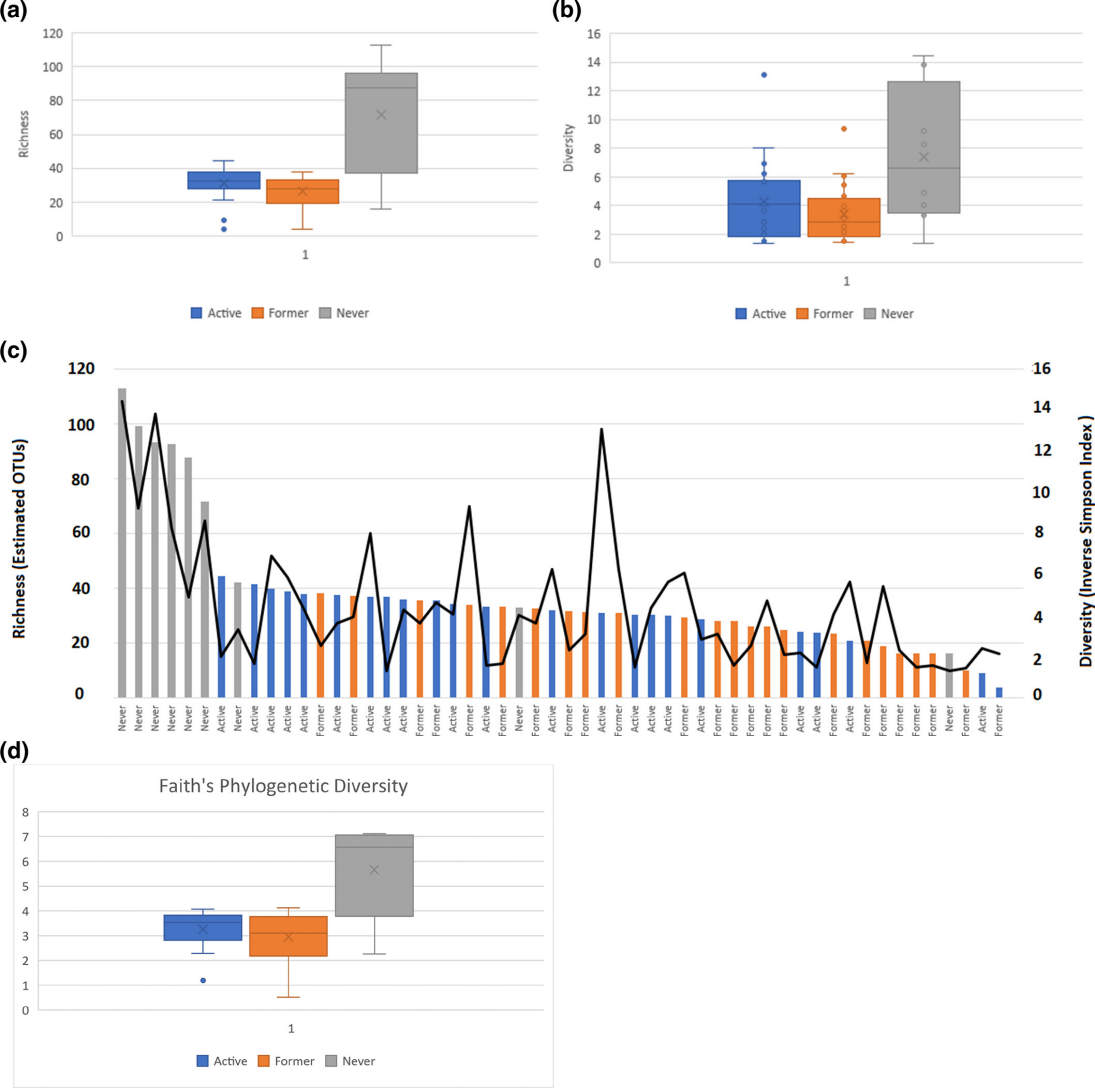
Ecological analytical measures of the LRT microbiome based on smoking status. (a) OTU richness computed using the Chao index. (**b)** Diversity is shown as the inverse Simpson diversity index. (**c)** The diversity plot (black line) and the richness bar graph (coloured vertical bars) with subjects ordered by decreasing richness values. (**d)** Faith’s phylogenetic diversity index.

Ecological analytical measures routinely used in human microbiome studies were also used for analysis. OTU richness and diversity were computed using the Chao [32] and inverse Simpson [33] diversity indices, respectively. NS exhibited markedly higher average richness (ANOVA [46], *P*=1e-9) and diversity (ANOVA, *P*=0.00075) compared to FS and AS (Fig. 4a, b), within errors for richness, although diversity had a wider error bar. This was more evident when the subjects were ordered by decreasing richness (Fig. 4c), suggesting that NS had higher richness. However, the diversity measured using the inverse Simpson index showed only a moderate correlation with the richness values and the smoking status (Fig. 4c). Table 4 shows Pearson correlations between richness and diversity along with associated *P*-values across all samples, and within each group. Interestingly, the NS showed the highest (0.75) and most statistically significant (*P*=0.02) correlation between richness and diversity, with the FS showing a weaker correlation (0.4) that is still biologically significant (*P*=0.05). The AS experienced almost no correlation at all (0.09, *P*=0.69). Across the entire set, a statistically significant (*P*=3e-7) positive correlation of 0.63 was observed between richness and diversity. Faith’s phylogenetic diversity index [47] also showed a higher value within errors for NS (ANOVA, *P*=2e-8) compared to AS and FS (Fig. 4d).

**Table 4.** Correlations between richness and diversity. Pearson correlations for richness (measured using the number of OTUs) and diversity (measured using the inverse Simpson index) for the entire set of samples, and the active, former and never smoker datasets

Samples	Pearson correlation	*P*-value
Entire set	0.63	3e-7
Active	0.09	0.69
Former	0.4	0.05
Never	0.75	0.02

### Biomarker analyses

**Fig. 5.**
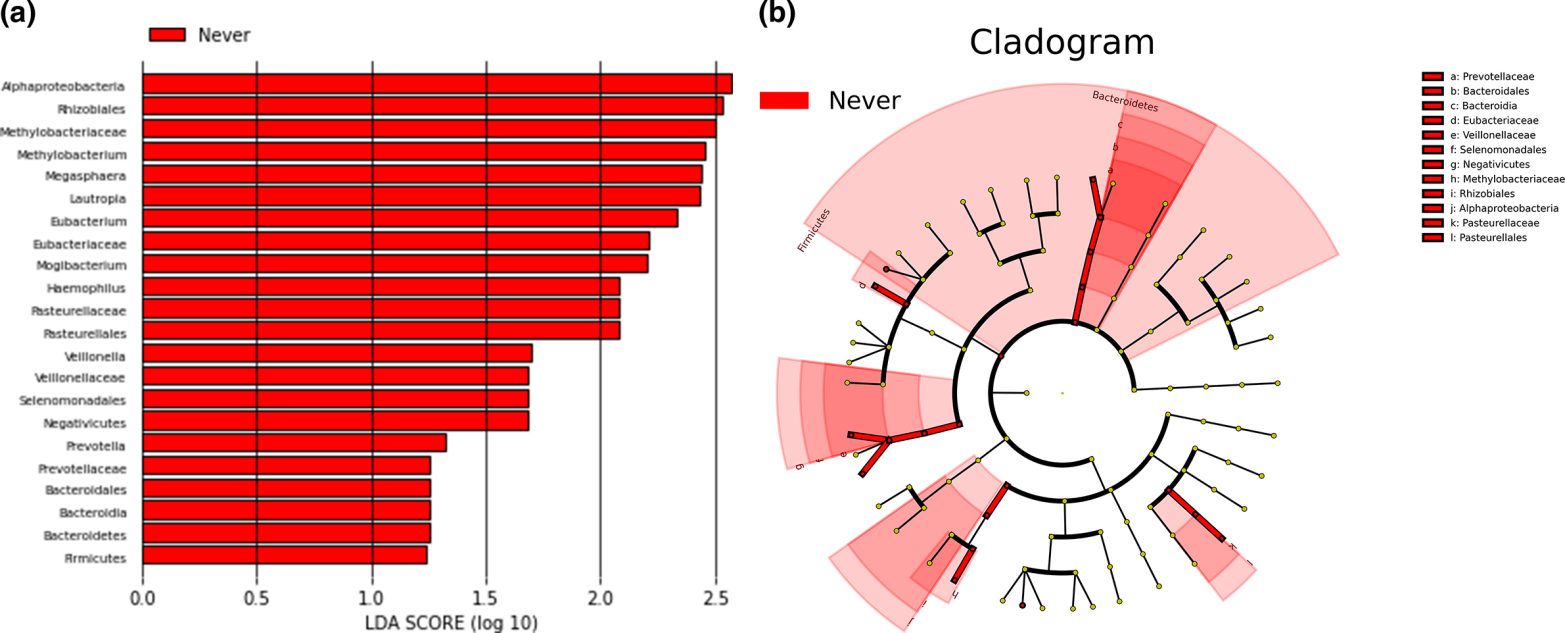
Biomarker analysis. Linear discriminant analysis effect size analysis (LEfSe) was used to identify the classes of bacteria contributing significantly to the differences observed within the different groups. The microbiome difference is represented as a cladogram (**a**) and the scores as a histogram (**b**). (a) Taxonomy nodes in the cladogram are shown as nested sectors arranged from the top level of the hierarchy at the centre and progressing outward. Taxonomic representation of statistically and biologically consistent differences between active, former and never smokers, although only the never smokers had distinguishing taxa. (b) The LDA scores of the top genera showing significant (LDA score threshold >1) differences between smoking groups are reported. For this particular group, only never smokers had distinguishing taxa.

Next, we used the LEfSe to identify the classes of bacteria contributing significantly to the differences observed between the three cohorts [35]. LEfSe even takes into account the low-abundance taxa in a sample used for the identification of potential biomarkers (*P*<0.05, LDA effect size >1) to produce a set of taxa with abundances most significantly different between the cohorts (Fig. 5). The cladogram (Fig. 5a) and the histogram with the LDA scores (Fig. 5b) show taxa that are statistically and biologically differential between the three groups. While our earlier compositional analysis provided feedback on differences between highly abundant taxa, LEfSe can be helpful for discovering less abundant taxa that tended to only occur in one of the three groups, and considers their taxonomic relationships. Interestingly, only the NS group came away with any distinguishing taxa, including the genera *Prevotella *, *Veillonella *, *Haemophilus *, *Mogibacterium *, *Eubacterium *, *Lautropia *, *Megasphaera* and *Methylobacterium *. Five of these – *Prevotella *, *Veillonella *, *Haemophilus *, *Lautropia* and *Methylobacterium* –were also uncovered by DESeq2 as distinguishing the NS samples. *Eubacterium* [45] has previously been reported as part of a healthy oral microbiome. We also see phyla – Firmicutes and Bacteroides, which makes sense given our earlier results with the box-and-whisker plots – where Proteobacteria was much more dominant in the AS and FS samples compared to NS at the expense of both Firmicutes and Bacteroides.

Table 5 shows differentiating taxa identified using LEfSe, ordered by taxonomic classification. All of these were reported as biomarkers for the NS group.

**Table 5.** Taxa identified in the LEfSe analyses

Group	Phylum	Class	Order	Family	Genus
**Never**	Proteobacteria	Alphaproteobacteria	Rhizobiales	* Methylobacteriaceae *	* Methylobacterium *
	Proteobacteria	Betaproteobacteria	Burkholderiales	* Burkholderiaceae *	* Lautropia *
	Proteobacteria	Gammaproteobacteria	Pasteurellales	* Pasteurellaceae *	* Haemophilus *
	Bacteroidetes	Bacteroidia	Bacteroidales	* Prevotellaceae *	* Prevotella *
	Firmicutes	Clostridia	Clostridiales	* Clostridiales */*incertae sedis*	* Mogibacterium *
	Firmicutes	Clostridia	Clostridiales	* Eubacteriaceae *	* Eubacterium *
	Firmicutes	Negativicutes	Veillonellales	* Veillonellaceae *	* Veillonella *
	Firmicutes	Negativicutes	Selenomonadales	* Selenomonadaceae *	* Megasphaera *

### Discriminant analyses

**Fig. 6.**
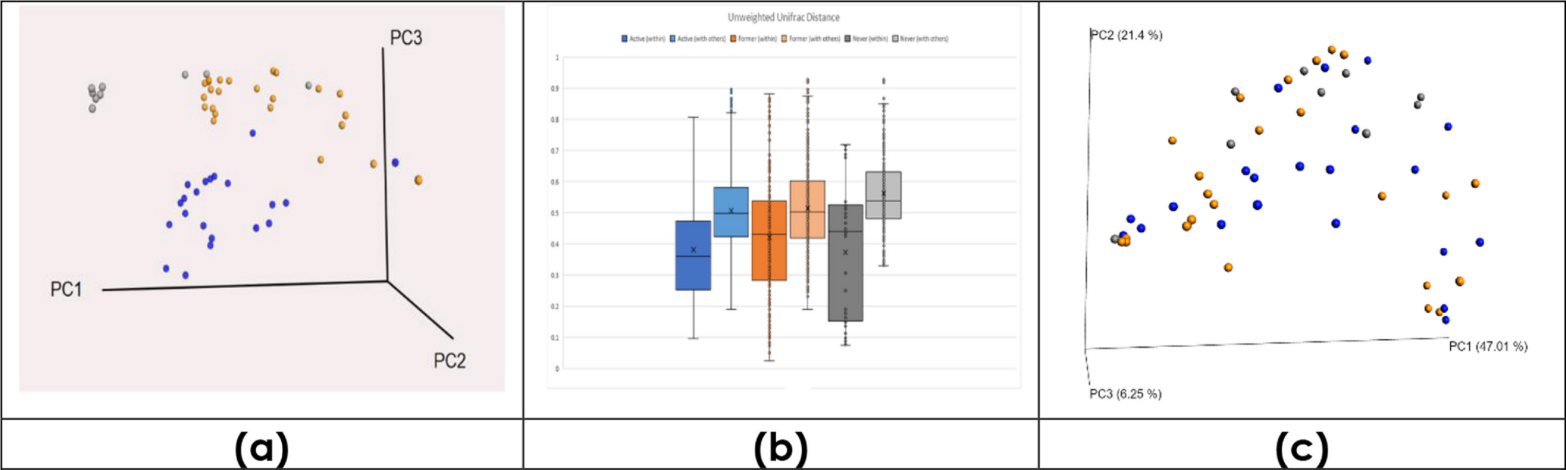
Clustering analyses of the LRT microbiome of the active, former and never smokers. (a) 3D principal coordinate analysis (PCoA) between groups, using unweighted Unifrac distance. (**b)** For each group, unweighted Unifrac distance between samples within that cohort, and samples within and those outside. (**c)** 3D PCoA between groups, using weighted Unifrac distance.

To determine if dimensionality reduction of the data points can lead to separation of the three groups,

PCoA [36] was performed using unweighted Unifrac distance as the measure of beta diversity [48]. The separation between the three clusters (Fig. 6a), the FS, AS and NS, was clear, supporting a claim of compositional differences in the LRT due to smoking. This separation was confirmed by PERMANOVA [49], with a *P*-value of 0.017. Fig. 6b supports that the NS group was more differentiated than the other two groups based on unweighted Unifrac distance, although more variance can be seen in the AS group, which was confirmed by BetaDisper [50], with an average distance-to-median of 0.36 compared to 0.29 for FS and NS. When we move to weighted Unifrac (Fig. 6c), the separation is not as clear, indicating that rarer members of the microbiome may potentially be more distinguishing between the three sample sets. Collectively, the genera reported by LEfSe for distinguishing NS samples (* Prevotella *, *Veillonella *, *Haemophilus *, *Mogibacterium *, *Eubacterium *, *Lautropia *, *Megasphaera* and *Methylobacterium *) comprise on average 19.1 % of the relative abundance of those samples, offering some additional support for this claim.

**Fig. 7.**
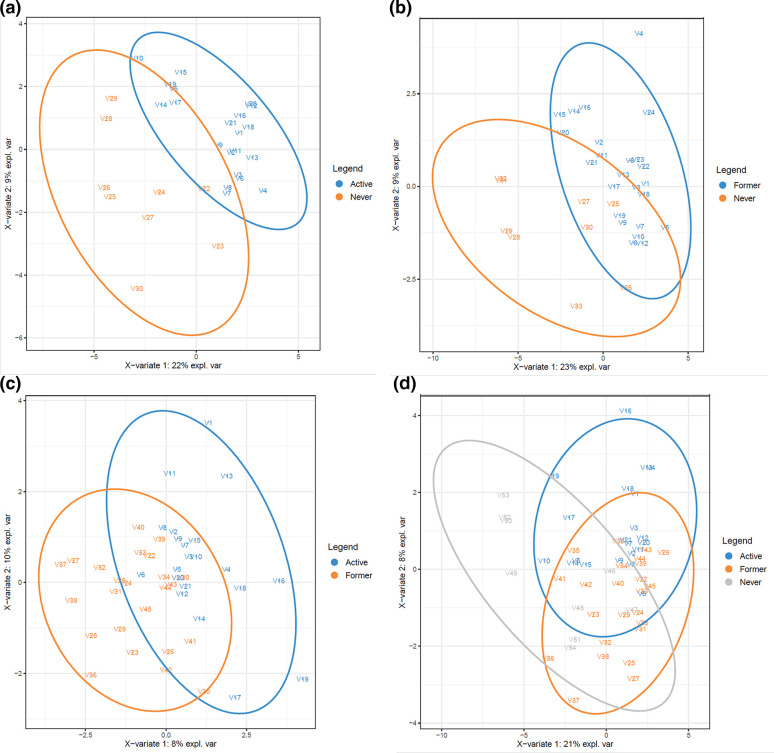
Discriminant analysis of microbiome profiles. A bilinear factor model, the partial least square discriminant analysis (PLS-DA) method, was used for differentiating between (**a**) active and never smokers, (**b**) former and never smokers, (**c**) active and former smokers, and (**d**) the three groups – active, former and never smokers – simultaneously. The explained variation in the plots (from the two variates) and the cross-validation errors are as follows: (**a**) 22 %+9 %, CV=0.2; (**b**) 23 %+9 %, CV=0.25; (**c**) 8 %+10 %, CV=0.4; (**d**) 21 %+8 %, CV=0.5.

To understand the differential relative abundance of the OTUs within the three study groups, we utilized a bilinear factor model, the partial least square discriminant analysis (PLS-DA [37]) method, which finds two (or more) composite directions (variates) that best separate the groups (Fig. 7). Again, clear separations were observed between the NS and AS groups (Fig. 7a) and the NS and FS groups (Fig. 7b). The separations between the AS and FS groups were less clear (Fig. 7c), also supported by the fact that the FS group showed overlap with both NS and AS clusters, when all three groups were analysed simultaneously (Fig. 7d). M-fold cross-validation (CV) errors were also reported in the legend, indicating the mean-squared error of prediction.

### Correlation between OTUs

**Fig. 8.**
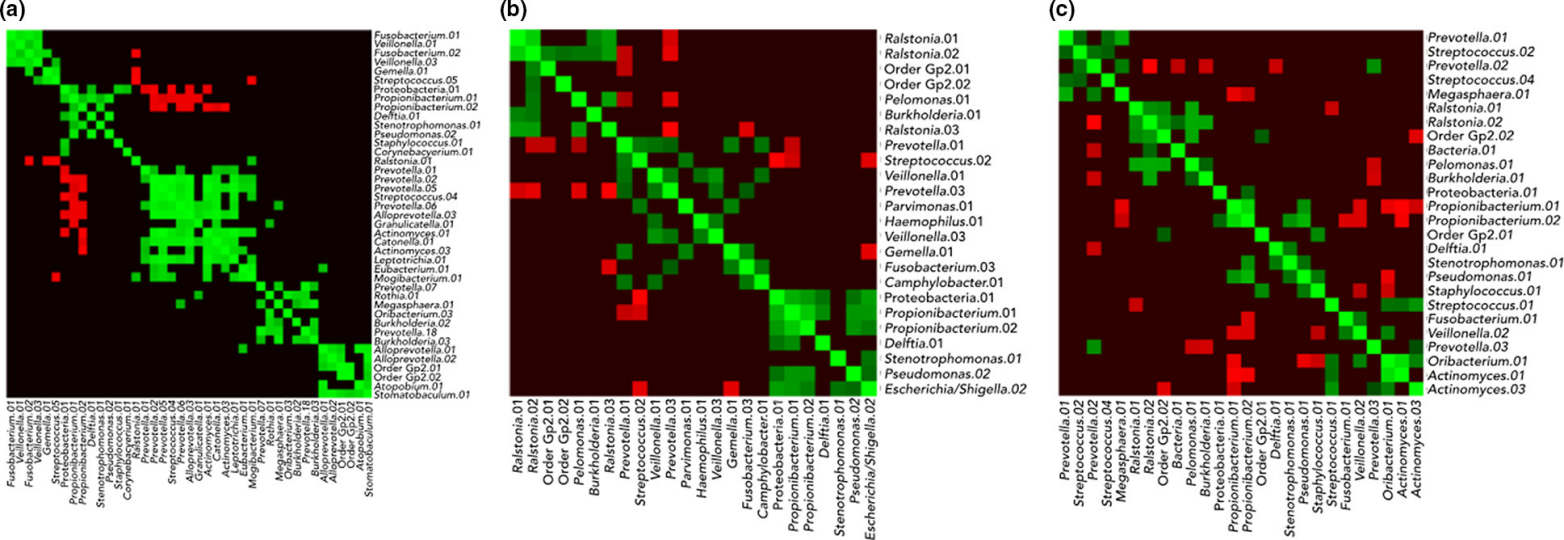
Correlations of vector abundance between OTUs. SparCC correlations between the OTU relative abundance vectors were calculated and visualized as a heatmap after clustering with the unsupervised Markov cluster algorithm was used for the (**a**) NS, (**b**) FS and (**c**) AS groups. The green and red colours refer to positive and negative correlations, respectively. The bright green square regions along the diagonal represent clusters, and the brightness indicates the tightness of the associations. The OTU clusters identified are detailed in Table 6.

The matrix of correlations (computed using SparCC [39]) between the vectors of relative abundance values of OTUs within each subject group was analysed using a fast and scalable unsupervised cluster algorithm called the Markov cluster algorithm (MCL) [38]. A heatmap was constructed from the matrix with green and red colours for positive and negative correlations, respectively (Fig. 8). The bacterial OTUs were then clustered using the correlation values as the distance function. The heatmap was reorganized by permuting rows and columns such that those corresponding to the same cluster of OTUs were located next to each other. The bright green square regions along the diagonal represent clusters, and the brightness indicates the tightness of the associations. The output shows that there is detectable clustering of OTUs in each of the three cohorts. The NS heatmap reveals tighter clusters with one large one (Fig. 8a). The FS clusters (Fig. 8b) appear to be better formed compared to the AS group, where the clusters are smaller and more weakly associated (Fig. 8c, Table 6).

**Table 6.** Distinctive OTU clusters (clubs) observed in each of the subject groups, and the average correlations within each cluster*. The number next to each cluster represents the mean strength of association between OTUs in that particular cluster, from 0 to 1 (strongest). Cluster colours correspond to those used in the networks (Fig. 9)

Never smokers	Former smokers	Active smokers
**A (0.83)**	**A (0.62)**	**A (0.63)**
P.Proteobacteria.01	P.Proteobacteria.01	P.Proteobacteria.01
* Propionibacterium *.01	* Propionibacterium *.01	* Propionibacterium *.01
* Propionibacterium *.02	* Propionibacterium *.02	* Propionibacterium *.02
* Delftia *.01	* Delftia *.01	* Delftia *.01
* Stenotrophomonas *.01	* Stenotrophomonas *.01	* Stenotrophomonas *.01
* Pseudomonas *.02	* Pseudomonas *.02	* Pseudomonas *.02
* Staphylococcus *.01	* Escherichia */* Shigella *.01	* Staphylococcus *.01
*Carynebacterium*.01	**B (0.62)**	**B (0.64)**
**B (0.89)**	* Ralstonia *.01	* Ralstonia *.01
* Alloprevotella *.01	* Ralstonia *.02	* Ralstonia *.02
* Alloprevotella *.02	O.Gp2.02	O.Gp2.02
O.Gp2.01	*Pelamonas*.01	*Pelamonas*.01
O.Gp2.02	* Burkholderia *.01	* Burkholderia *.01
* Atopobium *.01	O.Gp2.01	K.Bacteria.01
* Stomatobaculum *.01	* Ralstonia *.03	**C (0.55)**
**C (0.89)**	**C (0.54)**	* Prevotella *.01
* Ralstonia *.01	* Prevotella *.01	* Streptococcus *.02
* Prevotella *.01	* Streptococcus *.01	* Prevotella *.02
* Prevotella *.02	* Veillonella *.01	* Streptococcus *.04
* Prevotella *.05	* Prevotella *.03	* Megasphaera *.01
* Streptococcus *.04	* Parvimonas *.01	**D (0.59)**
* Prevotella *.06	* Haemophilus *.01	* Streptococcus *.01
* Alloprevotella *.03	* Veillonella *.03	* Fusobacterium *.01
* Granulicatella *.01	* Gemella *.01	* Veillonella *.02
* Actinomyces *.01	* Fusobacterium *.03	* Prevotella *.03
* Catonella *.01	*Camplyobacter*.01	* Oribacterium *.01
* Actinomyces *.03		* Actinomyces *.01
* Leptotrichia *.01		* Actinomyces *.03
* Eubacterium *.01		
* Mogibacterium *.01		
**D (0.87)**		
* Fusobacterium *.01		
* Veillonella *.01		
* Fusobacterium *.02		
*Veilonella*.03		
* Gemella *.01		
* Streptococcus *.05		
**E (0.84)**		
* Prevotella *.07		
Rothia.01		
* Megasphaera *.01		
*Oribacerium*.03		
* Burkholderia *.02		
* Prevotella *.18		
* Burkholderia *.03		
Negative associations between bacterial groups (clusters)^*^		
**Never smokers**	**Former smokers**	**Active smokers**
**A–C (−0.83)**	**B–C (−0.55)**	**A–D (−0.58)**
		**B–C (−0.57)**

*Average correlations for the edges between strongly rival clusters (clubs).

### Bacterial co-occurrence networks

**Fig. 9.**
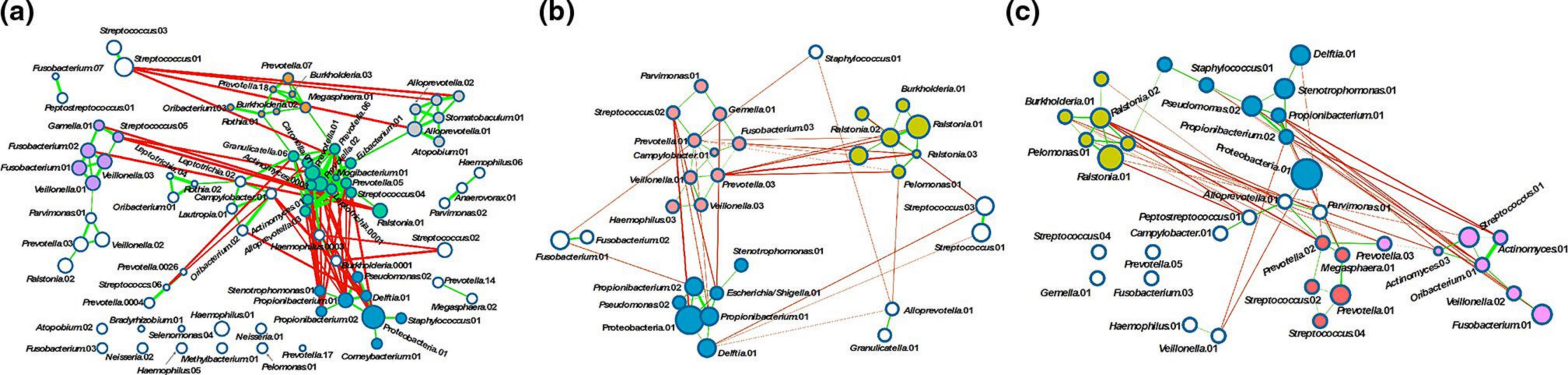
Construction of microbial co-occurrence networks. Proclivities of OTUs to co-occur (or co-avoid) in each of the subject groups, (**a**) NS (**b**) FS and (**c**) AS, are visualized as network diagrams constructed from pairwise correlation (SparCC [39], *P*=0.05) matrices using Cytoscape. Each node corresponds to an individual OTU. The sizes of the nodes are proportional to the relative abundance of the taxa they represent. The edges are coloured green or red depending on whether the taxa co-habit (positively correlated, as in a ‘social club’ [26]) or co-avoid (negatively correlated, as in ‘rival clubs’). The thickness of an edge is indicative of the strength of the correlation, with stronger associations represented by thicker edges. The Fruchterman–Reingold algorithm [41] was used for visualization, making the location of a node with respect to its neighbours indicative of its co-occurrence strength with that neighbour. Nodes are coloured by clusters.

To estimate the ecological relationships [26, 51] between the microbes in these samples, we constructed a microbial co-occurrence network (CoN) as described previously [26]. When visualized as a network (Fig. 9), the distinct clusters of OTUs that tended to co-occur together within each subject group were distinguishable. An overall comparison of the OTU networks showed that the NS clusters were tighter (thick and shorter edges; Fig. 9a). The OTU clusters observed in AS subjects were weaker (thinner and longer edges) (Fig. 9c), suggesting a weaker overall community structure. Quantification of OTU co-occurrence confirmed the ‘tightness’ in the NS clusters (correlation coefficient=0.79) compared to those in smokers (0.46 and 0.45 in FS and AS, respectively) (Table 7). The number of social clubs was higher in NS (5) compared to FS (3) and AS (4). AS had two rival club pairs, while NS and FS each had only one (Table 7).

**Table 7.** Number of distinctive clusters (clubs) in each population. Determined in the microbial co-occurrence network with social and rival clubs referring to positive and negative correlations, respectively

	No. of clubs (correlation coefficient ±sd with *P*<0.05)		
**Clubs**	**Never smokers**	**Former smokers**	**Active smokers**
**Social**	5 (0.79±0.12)	3 (0.46±0.17)	4 (0.45±0.18)
**Rival**	1 (−0.83±0.03)	1 (−0.55±0.07)	2 (−0.57±0.07)

The compositions and strength of associations of the most prominent OTU clusters detected in each subject group are detailed in Table 7 (with clusters named arbitrarily and not meant for comparison between subject groups). It is noteworthy that one cluster is shared across all three groups (cluster A), distinguished only by the presence of *Staphylococcus* in AS and NS groups, *Escherichia */* Shigella* in the former group, and *Corynebacterium* in the NS group. Active and former smokers also share a similar cluster in their respective cluster B, distinguished by the strong *Ralstonia* presence. The largest group with the strongest correlation in NS, group C, is largely dominated by *Prevotella* and *Actinomyces *, plus a *Streptococcus* OTU. Taxa from these genera also co-occurred within the other two groups, just within smaller and less tight-knit communities. Rivalries in AS occur between clusters B (heavy *Ralstonia *) and C (heavy *Prevotella */* Streptococcus *), and also clusters A (high phylum Proteobacteria and *Propionibacterium *) and D (two *Actinomyces* taxa, plus *Prevotella *, *Streptococcus* and *Veillonella *). The FS cluster B (also high *Ralstonia *) rivalled its cluster C (distinguished by *Prevotella* and *Veillonella *). The single rivalry in the NS network was between cluster A (Proteobacteria/* Propionibacterium *) and C (* Prevotella */* Actinomyces *, and a single *Streptococcus *).

*Ralstonia* was earlier reported in our Krona charts as much more abundant in FS and AS groups compared to NS, and all three *Ralstonia* taxa were reported by DESeq2 as distinguishing FS and AS samples compared to NS. Here, we see a nearly identical *Ralstonia *-heavy cluster of taxa forming in the FS and AS networks, in both cases rivalling a cluster consisting of many previously reported healthy oral and lung microbes, including *Streptococcus *, *Veillonella *, *Prevotella *, *Haemophilus* and *Fusobacterium *. All five of these taxa were reported by DESeq as distinguishing the NS group (three by LEfSe as well), which leads to questions regarding the potential impact of *Ralstonia* in the FS and AS microbial ecosystems. One other important observation is that a small community of co-occurring bacteria (cluster A) were found to be common among all three groups. Possible explanations of this phenomenon could that these bacteria may be part of a ‘core’ microbiome playing an important functional role in the lung.

Note that as we performed 16S analysis using OTU clustering, we kept all of our analyses at the genus level, where this method has previously been illustrated to be most accurate. As a reference, however, we took the representative sequence of every OTU and ran blast [52] against the database of genomes from its corresponding genus. Table 8 shows the closest matching species and strain for every one of our taxa.

**Table 8.** Closest matching species and strain for each OTU. Determined by running blast against sequences for the corresponding OTU genus. Two had no match, for *Stomatobaculum* (*) only one species is known (provided)

Genus-level taxon	Species	Strain
*Actinomyces.*01,03	* Actinomyces vulturis *	VUL7
*Alloprevotella.*01,03	* Alloprevotella rava *	DSM22548
*Alloprevotella.*02	* Alloprevotella tannerae *	ATCC51259
*Anaerovorax.*01	No match	No match
*Atopobium.*01	* Atopobium fossor *	DSM15642
*Atopobium.*02	* Atopobium minutum *	10 063 974
*Bradyrhizobium.*01	*Bradyrhizobium aeschynomenes*	83 002
*Burkholderia.*01	*Burkholedria pseudomultivorans*	BCC1191
*Burkholderia.*02	*Burkholderia perseverans*	INN12
*Burkholderia.*03	* Burkholderia puraquae *	LMG29660
*Campylobacter.*01	*Campylobacter troglodytis*	MIT059149
*Catonella.*01	*Catonella massiliensis*	Marseille-Q4567
*Corynebacterium.*01	* Corynebacterium phocae *	M408/89/1
*Delftia.*01	* Delftia lacustris *	FDAARGOS890
*Escherichia/Shigella.*0001	* Escherichia ruysiae *	C61-1
*Eubacterium.*01	* Eubacterium xylanophilum *	ATCC35991
*Fusobacterium.*01	* Fusobacterium canifelinum *	FDAARGOS1126
*Fusobacterium.*02	* Fusobacterium perfoetens *	ATCC29250
*Fusobacterium.*03	*Fusobacterium moriferum*	NZ
*Fusobacterium.*07	*Fusobacterium variums*	ATCC27725
*Gemella.*01	*Gemella sanguninis*	FDAARGOS742
*Granulicatella.*01	*Granulicatella rosea*	DSM18704
*Haemophilus.*01	* Haemophilus parainfluenzae *	ATCC33392
*Haemophilus.*03	* Haemophilus seminalis *	SZYH2
*Haemophilus.*05	* Haemophilus parahaemolyticus *	FDAARGOS1199
*Haemophilus.*06	* Haemophilus sputorum *	HK2154
K.Bacteria.01	*Clostridium phoceensis*	GD3
*Lautropia.*01	* Lautropia mirabilis *	NCTC12852
*Leptotrichia.*01	* Leptotrichia shahii *	JCM16776
*Leptotrichia.*02,04	* Leptotrichia wadei *	JCM16777
*Megasphaera.*01–02	* Megasphaera stantonii *	AJH120
*Methylobacterium.*01	* Methylobacterium isbiliense *	DSM17168
*Mogibacterium.*01	* Mogibacterium timidum *	ATCC33093
*Neisseria.*01	*Neisseria brasiliensis*	N17716
*Neisseria.*02	*Neisseria elongate*	M15911
O.Gp2.01	* Veillonella dispar *	NCTC11831
O.Gp2.02	*Gloebacter morelensis*	MG62769
*Oribacterium.*01,03	* Oribacterium parvum *	ACB1
*Oribacterium.*02	* Oribacterium asaccharolyticum *	ACB7
*Parvimonas.*01–02	* Parvimonas parva *	S3374
*Pelomonas.*01	* Pelomonas saccharophila *	DSM654
*Peptostreptococcus.*01	* Peptostreptococcus stomatis *	DSM17678
P.Proteobacteria.01	* Alcanivorax profundi *	MTE017
*Prevotella.*01–02, 04–05	* Prevotella bryantii *	TS1-5
*Prevotella.*03	* Prevotella nigrescens *	F0109
*Prevotella.*06	* Prevotella oryzae *	DSM17970
*Prevotella.*07,14	*Prevotella marseillensis*	Marseille-P8229
*Prevotella.*17	* Prevotella illustrans *	A2931
*Prevotella.*18	* Prevotella denticola *	F0115
*Prevotella.*26	* Prevotella herbatica *	WR041
*Propionibacterium.*01–02	* Propionibacterium freudenreichii *	PFRJS14
*Pseudomonas.*02	*Pseudomonas saudiphocaensis*	PS399
*Ralstonia.*01	* Ralstonia mannitolilytica *	SN82F48
*Ralstonia.*02	* Ralstonia pseudosolanacearum *	RS476
*Ralstonia.*03	* Ralstonia syzygii *	LLRS-1
*Rothia.*01	* Rothia koreensis *	JCM15915
*Rothia.*02	* Rothia aerolata *	CCM8669
*Selenomonas.*04	* Selenomonas noxia *	F0398
*Staphylococcus.*01	* Staphylococcus ratti *	CCM9025
*Stenotrophomonas.*01	* Stenotrophomonas rhizophila *	DSM14405
*Stomatobaculum.*01	Stomatobaculum longum*	(no match)
*Streptococcus.*01	* Streptococcus gordonii *	NCTC10231
*Streptococcus.*02	* Streptococcus phocae *	ATCC51973
*Streptococcus.*03	* Streptococcus mitis *	OT25TZ90
*Streptococcus.*04	* Streptococcus iniae *	9117
*Streptococcus.*05	* Streptococcus chenjunshii *	Z15
*Streptococcus.*06	* Streptococcus sobrinus *	10 919
*Veillonella.*01–02	* Veillonella criceti *	NCTC12020
*Veillonella.*03	* Veillonella ratti *	ATCC17746

## Discussion

This study aims to understand the LRT microbiome’s organization and explore changes that may relate to smoking status beyond looking at just the abundance and diversity.

The presence of OTUs characteristic of oropharyngeal samples in BAL has been a universal finding even among groups that have taken multiple steps to maximize sterility [13, 25, 53]. Direct tissue sampling from surgical specimens has confirmed the presence of oropharyngeal species [16, 19]. Using a neutral model of community ecology, Morris *et al.* concluded that dispersal from the mouth is mostly responsible for the lung’s microbial composition [25]. It has been reported that the upper airway microbiome resembles that of the oral cavity more than the microbiome of more distal airways, suggesting that there may be a gradient of aspirated bacteria [18]. BAL samples dominated by bacteria typically found in oral microbiomes are associated with an elevation of inflammatory markers [54]. Our study shows that healthy nonsmoker BALs have significantly higher relative abundances of several classic LRT microbes, such as *Prevotella *, *Streptococcus* and *Veillonella* (Fig. 3), as previously described [10, 16, 25]. Other known oral microbes [45] were also found, including *Neisseria* in former smokers and *Haemophilus *, *Leptotrichia* and *Fusobacterium* in nonsmokers. Some of our results matched those of Morris *et al.* [25] for nonsmokers, with elevated *Prevotella *, *Streptococcus *, *Veillonella *, *Fusobacterium* and *Haemophilus* (NS) and *Neisseria* (FS). Their study also found several other genera to be differentially abundant in nonsmokers’ lungs (both former and never). Differences in our findings can be attributed to the choice of the region of the 16S rRNA gene utilized in recovering the metagenome [55]. Regardless of their origin, these bacterial species may be worth further exploration, as they can potentially play an important role as local immune system regulators, similar to commensal bacteria in other body niches [24, 56, 57].

### Role of *Ralstonia*

Several studies have addressed the impact of smoking on the microbiome of the nasopharyngeal and oral cavities, but only one specifically addressed the effect of smoking on the human LRT microbiome [25]. Morris *et al.* detected disruption of oral microbial communities but found no significant differences in the BAL microbiome or OTU profiles between nonsmokers and healthy smokers [25]. Our study indicates the smoking groups can be differentiated using general ecological measures such as richness and (to a lesser degree) diversity, as well as using PCoA, LEfSe and PLS-DA (Figs 5–7). In particular, Fig. 7 showed that the former smokers appeared to occupy an ‘intermediate’ state that showed overlap with both the active and never smokers, which illustrates that a more granular study for former smokers that accounts for variables such as the amount of time since quitting smoking, pack years, etc. might be relevant for future work.

In particular, the increased presence of multiple OTUs of *Ralstonia* in the LRT of active and former smokers, and the significant role they appear to play in the co-occurrence networks, is particularly noteworthy. *Ralstonia* OTUs have been noted as pathogens multiple times by the Centers for Disease Control and Prevention (CDC) [58–60] and have been implicated in infections in COPD [61] and cystic fibrosis patients [62]. Their increased relative abundance in the LRT of active and former smokers was at the expense of several core LRT taxa, including *Streptococcus *, *Prevotella* and *Veillonella *, as evidenced by the rival clubs in both networks between the *Ralstonia *-dominated cluster and clusters containing these core taxa. These co-avoidance relationships in the smokers’ networks may be interpreted as a disruption of the strong community structure observed in the nonsmokers’ network and may be signs of dysbiosis in the microbiomes of smokers’ LRT.

An accurate characterization of the human microbiome is paramount to appreciating its role in human health and disease and understanding the complexity of microbial systems [63, 64]. Microbes co-exist in communities with complex inter-species interactions, both positive and negative, that can affect the functional dynamics of their impact on the host via the virulence traits of some members [56, 65, 66]. The overall effects on the human host may represent the effects of the entire community rather than that of its individual members. Thus, the study of the human microbiome requires more in-depth microscale analysis to determine its complexity and variations with respect to health and disease, in order to provide a perspective at a macroscale level.

### Co-occurrence networks

Cluster analyses of the co-occurrence networks reveal groups of bacterial OTUs that tend to occur together, as well as groups that tend to co-avoid. Active smoking clearly alters the relative abundance of specific bacterial taxa and is associated with disruptions in the strong co-occurrence relationships between taxa found in healthy individuals. AS exhibited alternative clustering of co-occurring bacterial taxa, several of which contained genera with opportunistic pathogens.

### Shared microbes

In our co-occurrence networks we observe a potential ‘core’ group consisting of the following taxa: two OTUs from the genus *Propionibacterium *, and OTUs from *Delftia *, *Pseudomonas* and *Stenotrophomonas *. Possible explanations for this common cluster could be that these microbes play some housekeeping role in the lung or are involved in some processes and pathways unaffected by smoking.

### Community structure changes

By integrating and visualizing all the positive and negative associations of individual OTU pairs in a network, we observe mutualistic clusters in all subject groups. The ‘tighter’ alliances seen in nonsmokers’ LRTs may be indicative of greater eubiosis when compared to subjects exposed to tobacco smoke. We also found that several bacterial clusters exhibited a robust negative correlation (‘rival clubs’) in smoking groups, suggesting co-exclusion of these taxa. Rivalry may thus represent group co-avoidance, perhaps a form of dysbiosis, suggesting the likelihood of subtypes of microbiomes within the groups, which can be better elicited with larger study cohorts. Rivalry may also represent driving forces for competitive exclusions, which may favour important pathogenetic species [63]. Overall, descriptions of these complex ecological relationships are crucial, since much of what is known about microbiome–host immune interactions has come from the study of single bacterial pathogens. Future analyses with larger groups of healthy individuals from different geographical locations will further define the healthy complex microbial diversity of the lungs.

Smoking was associated with weakening bacterial cluster tightness and increased rivalries between clusters, which is more pronounced in the network for the active smokers than in the former smokers, suggesting that smoking plays a role in the lung community dynamics. It is not clear if the changes are due to a direct effect of cigarette smoke, or epiphenomena linked to the anatomical, physiological and immunological alterations associated with smoking. These alterations specifically pertain to immune/inflammatory changes in the host lung due to smoking [64], while numerous byproducts of host inflammation act as bacterial growth factors [57]. On the other hand, cigarette smoke induces inflammation partly via Toll-like receptor 9 (TLR9), which is activated by bacterial DNA [67]. The cycle of inflammation and bacterial growth has been described as central to the development of lung disease [68, 69]. For that reason, it has been advocated that therapies that reduce inflammatory parameters need to take into account the presence of persistent colonizers and host–bacterial interactions to be more effective [70].

LEfSe analysis was able to identify biomarkers for each group. Finding microbial biomarkers for smoking, which is a behavioural trait, is important since it may lead to interventions, therapies and treatments of diseases associated with such behaviours.

Although this is one of the largest studies using bronchoscopy samples on stable individuals, it still lacks the power to detect critical bacterial associations or measure smaller but essential differences in community structure. More extensive studies that incorporate whole-genome sequencing, amplicon sequence variants [71] and/or analysis integrating all 16S rRNA gene variable regions may improve the identification of individual species and help overcome these limitations [72]. High-throughput DNA molecular techniques are currently unable to detect bacterial viability, limiting conclusions regarding disease pathogenesis. The present study focused on bacteria, only one component of the total microbiome that encompasses viruses, fungi [73], archaea and other non-fungal eukaryotes, a limitation that can be overcome by alternative techniques such as whole-genome sequencing [74, 75]. Finally, this was a cross-sectional study and does not provide information about the long-term composition of the lung microbiome and its relation to disease susceptibility and progression. Incorporating time series analysis [76] and causality [77] is important for future work to increase the limited perspective provided by microbiome snapshots. Causality determination is challenging; however, the findings may help design future prospective studies that lead to a better mechanistic understanding of the smokers’ lung microbiome.

In conclusion, using comprehensive integrative analysis tools, we present a better characterization of the complex microbial communities of the LRT and how they change based on the host smoking status. It is clear that significant differences occur in the LRT microbiome due to smoking. It also appears that oral microbiota can seed the lung, especially in smokers, making an upper airway microbiome study also interesting for future work. Based on the PLS-DA analysis, former smoker microbiomes seemed to share properties observed in the lung microbiomes of both active smokers and non-smokers. Replication of these findings, followed by integration with next-generation analytical tools, will likely establish the impact of these microbial clusters on human health.
